# Active case-finding for tuberculosis by mobile teams in Myanmar: yield and treatment outcomes

**DOI:** 10.1186/s40249-017-0291-5

**Published:** 2017-06-02

**Authors:** Ohnmar Myint, Saw Saw, Petros Isaakidis, Mohammed Khogali, Anthony Reid, Nguyen Binh Hoa, Thi Thi Kyaw, Ko Ko Zaw, Tin Mi Mi Khaing, Si Thu Aung

**Affiliations:** 1 National Tuberculosis Programme, Corner of Mingyi Road and Minglar Street, Pathein township, Ayeyarwaddy Region Myanmar; 2 Department of Medical Research, Yangon, Myanmar; 3 Médecins sans Frontières, Operational Research Unit, Luxembourg, Luxembourg; 4 National Tuberculosis Programme, Hanoi, Vietnam; 5 Centre for Operational Research, International Union Against Tuberculosis and Lung Disease, Paris, France; 6 TB Unit, WHO Country Office for Myanmar, Yangon, Myanmar

**Keywords:** Mobile team, Active case finding, Chest X-ray, Treatment outcomes

## Abstract

**Background:**

Since 2005, the Myanmar National Tuberculosis Programme (NTP) has been implementing active case finding (ACF) activities involving mobile teams in hard-to-reach areas. This study revealed the contribution of mobile team activities to total tuberculosis (TB) case detection, characteristics of TB patients detected by mobile teams and their treatment outcomes.

**Methods:**

This was a descriptive study using routine programme data between October 2014 and December 2014. Mobile team activities were a one-stop service and included portable digital chest radiography (CXR) and microscopy of two sputum samples. The algorithm of the case detection included screening patients by symptoms, then by CXR followed by sputum microscopy for confirmation. Diagnosed patients were started on treatment and followed until a final outcome was ascertained.

**Results:**

A total of 9 349 people with symptoms suggestive of TB were screened by CXR, with an uptake of 96.6%. Of those who were meant to undergo sputum smear microscopy, 51.4% had sputum examinations. Finally, 504 TB patients were identified by the mobile teams and the overall contribution to total TB case detection in the respective townships was 25.3%. Among total cases examined by microscopy, 6.4% were sputum smear positive TB. Treatment success rate was high as 91.8% in study townships compared to national rate 85% (2014 cohort).

**Conclusions:**

This study confirmed the feasibility and acceptability of ACF by mobile teams in hard-to-reach contexts, especially when equipped with portable, digital CXR machines that provided immediate results. However, the follow-up process of sputum examination created a significant barrier to confirmation of the diagnosis. In order to optimize the ACF through mobile team activity, future ACF activities were needed to be strengthened one stop service including molecular diagnostics or provision of sputum cups to all presumptive TB cases prior to CXR and testing if CXR suggestive of TB.

**Electronic supplementary material:**

The online version of this article (doi:10.1186/s40249-017-0291-5) contains supplementary material, which is available to authorized users.

## Multilingual abstract

Please see Additional file 1 for translations of the abstract into the six official working languages of the United Nations.

## Background

Myanmar is a country in South-East Asia that is classified by the World Health Organization (WHO) as one of the 30 high Tuberculosis (TB) burden countries. The incidence and mortality of TB in Myanmar is estimated to be 369 and 53 per 100 000 population in 2014, respectively [1]. The National Tuberculosis Programme (NTP) was established in 1966 to lead the efforts of TB control in the country. The programme adopted and implemented the Directly Observed Treatment Short Course (DOTS) strategy in 1997 and the Stop TB Strategy in 2007. In 2006, Myanmar achieved the previously set WHO targets of detecting 70% of estimated cases and successfully treated 85% of detected cases [2].

However, despite the success achieved over the past 10 years, a nationwide TB prevalence survey conducted between 2009 and 2010 showed a higher TB prevalence in urban than rural areas (330 vs 216 per 100 000 population) and higher in states than in regions (369 vs 191 per 100 000 population) respectively [3]. In Myanmar, the regions are located in the middle part of the country and most areas are composed of plain areas and described as ethnically predominant while the states are ethnic minority-dominant and hilly. The survey also showed a higher TB prevalence (369 per 100 000 population) among ethnic minority groups in specific states [3]. These states are mostly rural and hard-to-reach areas. It is estimated that 100 out of 330 townships in Myanmar have limited access to health care services, both because of security concerns and geographical isolation [4]. The HIV prevalence among new TB patients was 8.5% in 2014 according to the nation-wide HIV sentinel surveillance survey [2]. The prevalence of multidrug-resistant (MDR) TB was 5% among new TB patients and 27.1% among previously treated TB patients as shown in the nation-wide drug resistant survey (2012-2013) [2]. Since its establishment, the NTP has mainly been relying on a passive case finding (PCF) strategy for detection of TB. However, in 2005, the programme started to implement small-scale active case finding (ACF) activities in 30 out of the 330 townships of Myanmar involving mobile team activities in those states with hard-to-reach areas. Nowadays, Myanmar NTP has increasingly implemented mobile team activities by using portable digital X-ray and sputum microscopy. This is also consistent with WHO recommended End TB Strategy which includes early diagnosis of tuberculosis and systematic screening of contacts and high risk groups as a component of first pillar: Integrated, patient-centred care and prevention [5].

The literature on ACF is mixed [6–8]. A large study carried out in India using ACF (screening of TB in households) resulted in the detection of a large number of presumptive pulmonary TB cases who were not accessed by the regular National TB programme with variation across different states [6]. However, a large community randomized trial in Zambia and South Africa as well as a recent systematic review and meta-analysis on the effects of ACF have shown that the benefits of earlier diagnosis on patient outcomes and transmission have not been established [7, 8]. A literature search identified only a limited number of studies on ACF from around the world, including few studies conducted in South Asian countries (Cambodia, India, and Taiwan Province of China); no published studies from Myanmar were identified. Thus, relevant accurate evidence regarding ACF is required to set up appropriate case finding policy and practice for NTP, Myanmar.

The aim of this study was to describe the contribution of the ACF strategy through mobile team activities, including screening with digital chest X-rays, detection of TB cases, characteristics of patients and treatment outcomes from October – December 2014.

## Methods

### Design

This was a descriptive study using routine programme data.

### General setting

Myanmar is a low-income country that is located in South East Asia and is bordered by Bangladesh, India, China, Laos and Thailand. According to the 2014 Population and Housing Census, the population is 51.4 million of whom approximately 70% resides in rural areas [9]. The country is divided administratively, into one Council Territory (Nay Pyi Taw), seven states and seven regions. The road infrastructure and transport system in Myanmar are weak, especially during the rainy season when it might take two-three days to travel from one township to another. The mountainous nature of some parts of country makes it difficult to access many villages, especially in the northern and eastern part of the country.

Health services in Myanmar are organized through three tiers (primary, secondary and tertiary health facilities). Primary and secondary health care services exist in all regions and states but tertiary ones are available only in some large regions (Yangon, Mandalay, Nay Pyi Taw and Magway). There are three to four districts in each region/state. Each district has four to five township hospitals, and there are one to two station hospitals and six to seven rural health centres in each township.

TB diagnostic services are available at all townships in the country and are also decentralized to some station hospitals. Treatment is provided at all townships, including rural health centres by decentralization of anti-TB drugs. TB services are integrated into primary health care and are provided free of charge. Diagnosis of pulmonary TB is mainly based on sputum smear microscopy, but also on clinical manifestations and chest X-ray result.

### Specific setting

The routine programme data of mobile team activity done in 20 townships in four regions and three states of Myanmar during October - December 2014 were analyzed in this study. These townships were purposely chosen to analyze treatment outcomes of diagnosed TB patients from mobile team activity. Approximately 2.4 million people are living in these 20 townships.

Township selection for mobile team activity was based on the high case load and hard-to-reach areas. Five townships were chosen from Yangon region, which is situated in Lower Myanmar and mainly composed of urban areas with 45 townships and a population of 6.1 million. Six townships were chosen from Magway, Sagaing and Mandalay regions located in Middle Myanmar, comprised of plateau and some hilly areas. Around 14 million people are living in 90 townships in these regions. Nine townships were chosen from three states located in Upper Myanmar, Kachin, Kayah and Shan, where ethnic minority groups are living in 35 townships with a population of 2.4 million.

### Mobile clinic activities

ACF relied on mobile teams that include at least eight members (a team leader, chest X-ray reader, two X-ray technicians, one laboratory technician, one counsellor, one nurse and a driver). Two to four weeks prior to the activity, the team leader went on a preparatory visit to the selected township to advertise the planned mobile team activities. Basic Health Staff (BHS) at the rural health centres in the villages of the township conducted a health education activity. Three to four days prior to the mobile team visit, the local authority informed the community about the date of the visit of mobile team. People above 15 years of age with cough more than two weeks, other respiratory symptoms and constitutional symptoms consistent with TB, as well as TB contacts, previously treated TB cases and MDR TB contacts were prioritized. However, children (under 15 years) with TB contact were also encouraged to come to selected sites [10].

People who came to the mobile site were firstly screened by BHS for TB symptoms. Socio-demographic data and risk factors for TB were collected from all symptomatic patients using a standard proforma. A chest X-ray (CXR) was taken, and if it was abnormal, two sputum samples, including one early morning and one spot specimen were collected. Sputum examination was also conducted for those with cough more than two weeks even if the CXR was normal. If TB was diagnosed by both CXR positive and sputum positive result or diagnosed by CXR positive, sputum negative and symptom highly suspicious of TB, anti-TB treatment was usually provided by TB co-ordinator of the respective township on the spot and also linked with respective BHS. TB patients were recorded in township TB register. Type of patient, type of disease and treatment regimen was classified according to WHO guideline [10]. The screening and diagnosing algorithm for TB in this study was quite similar to algorithm 2c of WHO [11]. HIV counselling and testing was done for all registered TB cases at the Township Health Department, not at the operation site. Similarly, GeneXpert testing was also recommended for retreatment cases, TB/HIV co-infected cases and MDR TB contacts, according to the diagnostic algorithm of NTP. However, GeneXpert testing could not be done at the operation site and the specimens were sent to District TB clinic where GeneXpert machine was set up.

### Definitions of TB treatment outcomes

The WHO definitions of TB treatment outcomes were used, as shown in Table 1 [12]. All bacteriologically confirmed and clinically diagnosed TB cases were assigned an outcome from this list except those with rifampicin resistant TB (RR-TB) or MDR TB, who were placed on a second-line drug regimen.

**Table 1:** WHO definitions of TB treatment outcomes

Outcome	Definitions
Cured	A pulmonary TB patient with bacteriologically-confirmed TB at the beginning of treatment who was smear or culture-negative in the last month of treatment and on at least one previous occasion.
Treatment completed	A TB patient who completed treatment without evidence of failure BUT with no record to show that sputum smear or culture results in the last month of treatment and on at least one previous occasion were negative either because tests were not done or because results were unavailable.
Treatment failed	A TB patient whose sputum smear or culture was positive at five months or later during treatment.
Died	A TB patient who died for any reason before starting or during the course of treatment
Lost-to-follow up	A TB patient who did not start treatment or whose treatment was interrupted for two consecutive months or more.
Not evaluated	A TB patient for whom no treatment outcome was assigned. This included cases “transferred out” to another treatment unit as well as cases for which the treatment outcome is unknown to the reporting unit.
Treatment success	The sum of cured and treatment completed

### Study population

All TB cases diagnosed through mobile team activity and started on treatment between October 2014 and December 2014 in 20 townships were included in the study.

### Data collection, variables and statistical analysis

Variables related to the study objectives were sourced from proforma, mobile team presumptive TB register, mobile team X-ray register and township TB register. They included age, sex, education status, TB contact history, CXR result, sputum result and its grading, type of disease, type of patient and treatment regimen. Data were entered from the paper-based proforma and registers by a trained data assistant of the mobile team into a data entry file created using EpiData Entry software (V.3.1. Odense, Denmark). The data files were checked by a responsible person, WHO TB unit, Country office for Myanmar. The patients detected and treated by either passive case finding or other alternative methods of active case finding were not recorded individually by using EpiData software. Data were analysed using EpiData analysis software (V.2.2.2.182. Odense, Denmark). We also calculated a number needed to screen (NNS) to detect a case of TB and defined as 1 per prevalence [11]. We used numerator as people with symptoms suggestive of TB and denominator as total number of any types of TB case identified (sputum-positive pulmonary TB, sputum-negative and EPTB).

## Results

### TB screening results

According to the model of ACF through mobile team used in this study, the community was announced in advance that people with symptoms suggestive of TB to come for screening of TB. So, almost all people who came to operation site were presumptive TB cases who were enrolled and interviewed first. Based on this number of presumptive TB cases, we calculated NNS and found that we need to screen 19 presumptive TB cases to get one case of any types of TB.

**Fig. 1:**
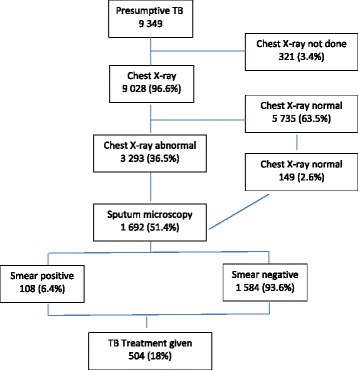
Flow chart of presumptive and diagnosed TB patients: mobile active case finding; Myanmar; October 2014 – December 2014

As per Table 2, between October and December 2014, a total of 9 349 presumptive TB patients were screened and of whom, 96.6% (9 028/9 349) received a chest X-ray (CXR). Thirty-six percent of those (3 293/9 028) had abnormal X-ray results and were meant to undergo sputum smear microscopy; however, only 51.4% (1 692/3 282) including 161 people with cough and normal CXR finally had a sputum examination (Fig. 1). Among these, 6.4% (108/1 692) patients were found to have positive sputum results.

**Table 2:** Number of presumptive TB cases examined by CXR and sputum microscopy through mobile team activity in four regions and three states, Myanmar, October 2014-December 2014

Region/State	Presumptive TB^a^ cases *n*	Evaluated with CXR^b^ *n* (%)	Persons with abnormal CXR *n* (%)	Evaluated with sputum microscopy *n* (%)	Smear positive cases & SPR^c^ *n* (%)
Yangon Region	2 010	2 009 (99.9)	727(36.2)	285 (39.2)	23 (8.1)
Mandalay Region	1 185	942 (79.5)	293(31.1)	65 (22.1)	3 (4.6)
Magway Region	1 171	1 167 (99.7)	465 (39.9)	209 (44.9)	6 (2.9)
Sagaing Region	1 073	1 073 (100)	745 (69.4)	347 (46.6)	20 (5.8)
Kachin State	2 555	2 485 (97.3)	732 (29.5)	494 (67.5)	39 (7.9)
Kayah State	671	669 (99.7)	85 (12.7)	72 (84.7)	0 (0.0)
Shan (East) State	684	683 (99.9)	246 (36.0)	220 (89.4)	17 (7.7)
Total	9 349	9 028 (96.6)	3 293 (36.5)	1 692 (51.4)	108 (6.4)

^a^ *TB* Tuberculosis

^b^ *CXR* Chest-X-Ray

^c^ *SPR* Sputum Positivity Ratio

Table 3 shows TB cases detected through mobile team activity and the contribution of ACF activity to the total TB cases in the study regions/states. There was a wide variation in this contribution, with the highest in Kayah and Shan (East) states, and lowest in Yangon and Mandalay regions. Overall, the average contribution was 25.3% (504/1 995).

**Table 3:** Contribution of TB case finding by mobile team activity to total TB case detection in four regions and three states, Myanmar, October 2014-December 2014

Region/State	Total TB^a^ cases detected (PCF^b^ + ACF^c^) *n*	TB cases detected by mobile team (ACF) *n*	Contribution %
Yangon Region	749	86	11.5
Mandalay Region	72	8	11.1
Magway Region	131	51	38.9
Sagaing Region	128	37	28.9
Kachin State	691	192	27.8
Kayah State	32	20	62.5
Shan (East) State	192	110	57.3
Total	1 995	504	25.3

^a^ *TB* tuberculosis

^b^ *ACF* active case finding

^c^ *PCF* passive case finding

### Characteristics of the study population

Among the 504 TB patients detected by mobile team activity, there were 393 (78.0%) with smear-negative pulmonary TB (PTB), 108 (21.4%) smear-positive pulmonary TB and three (0.6%) with extra-pulmonary TB. The demographic and clinical characteristics of the study population are provided in Table 4.

**Table 4:** Characteristics of tuberculosis patients diagnosed through mobile team activity in four regions and three states, Myanmar, October 2014-December 2014

Variable	*n* (%)
Total	504
Sex	
Male	309 (61.3)
Female	195 (38.7)
Age group (years)	
< 15	32 (6.3)
15-54	275 (54.6)
≥ 55	197 (39.1)
Median (IQR^a^)	49 (34-60)
Education status	
Illiterate	183 (36.3)
Read & write-Primary	195 (38.7)
Middle-High	115 (22.8)
University-Graduate	11(2.2)
History of TB^b^ contact	
No	399 (79.2)
Yes	99 (19.6)
Unknown	6 (1.2)
CXR^c^ result	
Normal	1 (0.2)
Highly suspicious of TB	485 (96.2)
Suspicious of TB	6 (1.2)
Healed TB	10 (2.0)
Others	2 (0.4)
Smear result and grading	
Negative	393 (78.4)
Scanty	7 (1.5)
1 +	40 (8.0)
2 +	26 (5.4)
3 +	35 (7.3)
Type of TB disease	
Smear-positive PTB^d^	108 (21.4)
Smear-negative PTB	393 (78.0)
EPTB^e^	3 (0.6)
Type of TB patient	
New TB case	448 (88.9)
Retreatment	
Relapse	
Treatment after Lost to follow-up	53 (10.5)
	3 (0.6)
TB treatment regimen	
Initial regimen (6 months)	416 (82.5)
Retreatment regimen (8 months)	56 (11.1)
Childhood regimen (6 months)	32 (6.3)

^a^IQR (Interquartile range), ^b^Tuberculosis, ^c^Chest X-ray, ^d^Pulmonary TB, ^e^Extrapulmonary TB

Treatment outcomes by type of TB disease, type of TB patient and by treatment regimen are shown in Table 5. The overall treatment success rate for all new TB cases was 93% (401/431) whereas the rates for death, loss-to-follow-up and failure were 2.3%, 2.3%, and 0.9%, respectively.

**Table 5:** Treatment outcomes of tuberculosis patients diagnosed through mobile team activity in four regions and three states, Myanmar, October 2014-December 2014

Variable	Total patients *n* (%)	Initial loss^a^ *n*	Treatment success_1_ *n* (%)	Failed *n* (%)	Died *n* (%)	LTFU^2^ *n* (%)	Not evaluated *n* (%)	Moved to SLD^3^ *n* (%)
All TB patients	504	20 (4.0)	438 (90.5)	7 (1.4)	13 (2.7)	13 (2.7)	9 (1.9)	4 (0.8)
New & Relapse	501	20 (4.0)	437 (90.5)	7 (1.5)	13 (2.7)	12 (2.5)	9 (1.9)	3 (0.6)
NSP PTB^4^	85	1 (1.2)	78 (91.8)	2 (2.4)	2 (2.4)	1 (1.2)	1 (1.2)	0 (0.0)
TB type								
SSP PTB^5^	108	2 (1.9)	93 (87.7)	4 (3.8)	3 (2.8)	2 (1.9)	1 (0.9)	3 (2.8)
SSN PTB^6^	393	18 (4.6)	342 (91.2)	3 (0.8)	10 (2.7)	11 (2.9)	8 (2.1)	1 (0.3)
EPTB^7^	3	0	3 (100)					
TB category								
New	448	17 (3.8)	401 (93.0)	4 (0.9)	10 (2.3)	10 (2.3)	6 (1.4)	0 (0.0)
Retreatment	56	3 (5.4)	37(66.0)	3 (6.3)	3 (6.3)	3 (6.3)	3 (6.3)	4 (7.1)
Relapse	53	3 (5.4)	36 (72.0)	3 (6.0)	3 (6.0)	2 (4.0)	3 (6.0)	3 (6.0)
LTFU^2^	3	0	1 (33.3)	0 (0.0)	0 (0.0)	1 (33.3)	0 (0.0)	1 (33.3)
Treatment								
IR^8^	416	15 (3.6)	371 (92.5)	4 (1.0)	10 (2.5)	10 (2.5)	6 (1.5)	0 (0.0)
RR^9^	56	3 (5.4)	37 (69.8)	3 (5.7)	3 (5.7)	3 (5.7)	3 (5.7)	4 (7.5)
CR^10^	32	2 (6.3)	30(100)	0 (0.0)	0 (0.0)	0 (0.0)	0 (0.0)	0 (0.0)

^1^Treatment Success includes patients who are cured and completed treatment

^2^LTFU: Loss-to-follow up; ^3^SLD: Second line drugs; ^4^NSP PTB: New smear positive PTB

^5^SSP PTB: Sputum smear positive Pulmonary TB ^6^ SSN PTB: Sputum smear negative Pulmonary TB; ^7^EPTB: Extra pulmonary TB ^8^IR: Initial Regimen ^9^RR: Retreatment Regimen ^10^CR: Childhood Regimen

^a^This is calculated on total TB patients diagnosed while other outcomes excluding initial loss from the denominators

## Discussion

This study of ACF, employing mobile teams using portable digital CXR demonstrated satisfactory yield and treatment outcomes. Almost all presumptive TB patients screened had a CXR and in one third it was abnormal. However, nearly half of patients with an abnormal CXR proceeded to have a sputum examination. This is important because it reinforces the routine use of CXR in screening for TB, but points out weaknesses in the collection of sputum for microbiological confirmation of the diagnosis. These results are encouraging for the implementation of ACF through mobile team activity more widely in Myanmar in order to meet the target of WHO’s End TB Strategy.

This is the first study of the use of portable digital X-rays for TB diagnosis, introduced into the field since 2013 in Myanmar. The high yield appears to justify its incorporation into future ACF activities, especially given its ease of use and immediate results. These findings are similar to use of CXR for screening in ACF as in Cambodia and India [7, 13]. Besides, systematic review also showed that CXR-based ACF programmes identify a significant proportion of active TB in vulnerable population [14]. Our NNS was very low as shown in the result, however, this is a limited calculation because we used already screened presumptive TB cases and very broad operational definition of TB cases including smear-positive pulmonary TB, smear-negative pulmonary TB and EPTB.

However, half of all those identified by abnormal CXR did not proceed to have a sputum examination to confirm diagnosis. This is similar to findings from the Axshya Project in India of active tuberculosis case finding in five million households where only 56% completed sputum examination following symptom screening [6].

The low uptake of sputum examination may be attributed to the following factors. First, the lack of one-stop-service for both CXR and sputum examination, with lab technician and microscope, meant that patients had to make a visit to the township to confirm diagnosis, thereby creating a disincentive to do so. Second, some patients presented with chest symptoms other than cough and could not produce sputum. Third, Basic Health Staff (BHS) may not have taken responsibility for transportation of sputum cups to the township hospital. Finally, there might be under recording of sputum examinations when the patients went to township hospital themselves or BHS transported sputum samples later after mobile activity.

Even though the contribution to total TB case detection was the lowest in Yangon region, the sputum positivity ratio was the highest. The high sputum positivity ratio might be due to sputum examination done only among those with highly suspicious of TB (among high risk groups such as diabetes, close contacts and old age, etc.), but not among all eligible persons or due to high prevalence of smear-positive TB in the community. This also pointed out that all eligible persons might be encouraged for sputum examination and mobile clinics might be one-stop service including sputum examination on the spot. In addition to this, it also highlighted that the model of ACF through mobile team by screening with symptoms and CXR followed by microbiological confirmation for TB was effective not only in hard-to-reach areas but also in urban, peri-urban and slum areas. In contrast to this, there was no sputum positive case in Kayah state. In this issue, quality-assured microscopy and dispersed population residing in hilly region might be the solutions, but not due to high HIV prevalence. Although the GeneXpert testing was recommended for all eligible persons, not all were examined. Besides, results were also missing in the records.

In terms of final outcomes, we found that 6.4% of patients screened with sputum microscopy were finally diagnosed with TB smear positive. This was similar to findings in the Axshya Project (8%) [6]. However, we noted that more patients were started on treatment on the basis of symptoms and CXR (78%) than sputum positivity (21%), again reinforcing the value of routine CXR. When compared to ACF group to Passive Case Finding (PCF) group in Cambodia, the similar finding was also noted [7]. The overall contribution of TB cases detected by mobile team activity to total TB case detection in respective townships was high at 25.3%.

The fact that 4% of TB cases detected and treated by the National TB programme in Myanmar came from accelerated case finding activities revealed that majority of the cases were from passive case finding in the national data [2]. The age and sex distribution of the patients detected through ACF are not very different between active case finding and national data (mostly by PCF). There are more male patients than female patients (1.6 : 1 vs 1.7 : 1) and more than half of patients were in the working age group (54.6% vs 51%). However, the proportion of initial regimen is higher in ACF than national data (82.5% vs 66%) and that of smear negative cases is also higher in ACF than national data (78% vs 65%). This is similar to other studies comparing ACF and PCF. And it also confirmed that ACF can potentially identify TB in an earlier stage of disease [15].

The results of treatment were encouraging, with an overall success rate of 90.5% and low initial loss (4%) and loss-to-follow-up (LTFU) from treatment (2.7%). These compare favourably with overall country-wide figures (85% treatment success and 5.3% LTFU) [2]. The initial loss was similar to a study in Cambodia (5%), and Zimbabwe (6%), in contrast, it was as high as a quarter of cases identified through screening in the South African and Indian studies [8]. The proportion of initial loss was quite acceptable because it was also found as 3% in ACF among 5 million household contacts in India [6]. In order to be zero initial loss, ACF through mobile team activity should be one-stop-service.

In this study, 23% reported history of TB contacts among overall presumptive TB cases. Of total contacts, 5% were diagnosed and started on treatment. In Karachi, Pakistan, 11.7% of household contacts had TB as a result of ACF [16]. Besides, among TB cases detected by the mobile team activity, only 19.6% reported a history of TB contact. Although it might be weakness in interview process and under recording, these findings reinforce the importance of encouraging all patients with symptoms, not just TB contact, to come for screening.

There were a number of strengths of this study. First, this was a first study in Myanmar to base its analysis of yield and treatment outcomes on individual patient data as opposed to aggregated data. Second, the coverage of the patients receiving CXR was virtually complete. Third, data entry was by trained assistants and quality checks were employed.

The study had some limitations. First this was an observational study and relied on routinely-collected data which may not have been entirely accurate. Second the study population was specifically chosen for areas of high TB caseload and hard-to-reach areas: thus, the study findings may not be generalizable to other parts of the country. Third, WHO’s new definitions on type of TB patients could not be used in this study because GeneXpert testing as well as Culture and Drug Susceptibility Testing (DST) could not be conducted for all eligible patients. We were not able to assess the burden of resistant form of TB among the screened population.

## Conclusion

Despite these limitations the study provided important insights regarding the feasibility and acceptability of an ACF strategy in this model conducted by mobile teams that may be employed and studied under operational conditions in various other regions and states in the country where the burden of TB is high and access to care is challenged. In order to optimize this strategy some additional measures should be considered especially universal access to microbiological examination as one stop service including molecular diagnostics or provision of sputum cups to all presumptive TB cases prior to CXR and only testing if CXR suggestive of TB.

## Additional file

Additional file 1: Multilingual abstract in the six official working languages of the United Nations. (PDF 655 kb)

## Abbreviations

ACF: Active case finding
BHS: Basic health staff
CXR: Chest X-ray
DOTS: Directly observed treatment short course strategy
EPTB: Extra pulmonary TB
HIV: Human immuno-deficiency virus
LTFU: Loss-to-follow up
MDR: Multi-drug resistant
NTP: National Tuberculosis Programme
PTB: Pulmonary TB
TB: Tuberculosis
WHO: World Health Organization

## References

[1] World Health Organization (2015). Global tuberculosis report 2015.

[2] National Tuberculosis Programme (2015). Annual Report 2014.

[3] National Tuberculosis Programme (2011). Report on National TB Prevalence Survey, 2009-2010.

[4] National Tuberculosis Programme (2013). Guideline for community based TB care to increase access to quality DOTS service.

[5] Executive Board, 134. Global Strategy and targets for tuberculosis prevention, care and control after 2015: Report by the Secretariat. 2014. http://www.who.int/iris/handle/10665/172828

[6] Prasad BM, Satyanarayana S, Chadha SS, Das A, Thapa B, Mohanty S (2016). Experience of active tuberculosis case finding in nearly 5 million households in India. Public Health Action.

[7] Eang MT, Satha P, Yadav RP, Morishita F, Nishikiori N, van-Maaren P (2012). Early detection of tuberculosis through community-based active case finding in Cambodia. BMC Public Health.

[8] Kranzer K, Afnan-Holmes H, Tomlin K, Golub JE, Shapiro AE, Schaap A (2013). The benefits to communities and individuals of screening for active tuberculosis disease: a systematic review. Int J Tuberc Lung Dis.

[9] Ministry of Health (2015). Health in Myanmar 2014.

[10] National Tuberculosis Programme (2014). Standard Operating Procedure on Mobile Team Activity 2014.

[11] World Health Organization (2015). Systematic screening for active tuberculosis: an operational guide.

[12] World Health Organization (2014). Definitions and reporting framework for tuberculosis-2013 revision.

[13] Binepal G, Agarwal P, Kaur N, Singh B, Bhagat V, Verma RP (2015). Screening difficult-to-reach populations for tuberculosis using a mobile medical unit, Punjab India. Public Health Action.

[14] Paquette K, Cheng MP, Kadatz MJ, Cook VJ, Chen W, Johnston JC (2014). Chest radiography for active tuberculosis case finding in the homeless: a systematic review and meta-analysis. Int J Tuberc Lung Dis.

[15] den Boon S, Verver S, Lombard CJ, Bateman ED, Irusen EM, Enarson DA (2008). Comparison of symptoms and treatment outcomes between actively and passively detected tuberculosis cases, the additional value of active case finding. Epidemiol Infect.

[16] Khan TR, Ahmed Z, Zafar M, Nisar N, Qayyum S, Shafi K (2014). Active case finding of sputum positive pulmonary tuberculosis in household contacts of tuberculosis patients in Karachi, Parkistan. J Assoc Chest Physicians.

