# Supplementary material for: Active case-finding for tuberculosis by mobile teams in Myanmar: yield and treatment outcomes
Source: Infect Dis Poverty. 2017 Jun 2;6:77. doi: 10.1186/s40249-017-0291-5 (PMC5455111; doi:10.1186/s40249-017-0291-5)
Supplement: Additional file 1: — Multilingual abstract in the six official working languages of the United Nations. (PDF 655 kb) [file 40249_2017_291_MOESM1_ESM.pdf]

Translation of the abstract into the six official working languages of the United Nations

عملية دراسة الحالات النشطة بشأن مرض السل من قبل المجموعات المتنقلة في ميانمار: النتائج الميدانية ونتائج المعالجة

أونمار ميانت، ساو ساو، بيتروس إيساكيديس، محمّد كوغالي، أنطوني رايد، نغوين بينهوا، تي تي كياو، كوك و زاو، تين مي مي كينغج، سي تو أونغ

ملخّص

تقديم عام: منذ سنة 2005، كان البرنامج الوطني لمكافحة السل في ميانمار **(NTP)** يقوم بأنشطة تتعلق بعملية دراسة الحالات **(ACF)** بمشاركة المجموعات المتنقلة في المناطق التي يصعب الوصول إليها. وقد كشفت هذه الدراسة عن مساهمة أنشطة المجموعات المتنقلة في الكشف عن حالات السل **(TB)**، كما كشفت عن خصائص مرضى السل الذين تمّ تحديدهم من قبل المجموعات المتنقلة فضلا عن نتائج علاجهم.

التقنيات المستخدمة: تم اعتماد دراسة وصفية باستخدام بيانات لبرنامج روتيني في الفترة الممتدة بين أكتوبر 2014 وديسمبر 2014. قدمت أنشطة المجموعات المتنقلة عديد الخدمات لتشمل التصوير الشعاعي الرقمي للصدر المحمول (CXR) والفحص المجهري لعينتين من البلغم. شملت عملية الكشف عن حالات الإصابة بالسل فحص المرضى استنادا إلى الأعراض ثم التصوير الشعاعي الرقمي للصدر وأخيرا للتأكد من النتيجة النهائية يخضع المريض لفحص مجهري لعينة من البلغم. بدأ المرضى المُصابون بالسل بأخذ العلاج وتمت متابعتهم للتأكد من النتيجة النهائية.

النتائج: خضع 9349 شخصا يُعانون من أعراض الإصابة بمرض السلّ للتصوير الشعاعي الرقمي للصدر مع امتصاص المغذيات بنسبة 96.6%. خضع 51.6 % للفحص المجهري لعينة من البلغم من بين أولئك الذين كان من المفترض خضوعهم لهذا الفحص. وأخيرا، تمّ تحديد 504 مُصابا بمرض السل من قبل المجموعات المتنقلة وقد بلغت نسبة الكشف عن حالات الإصابة بالسلّ في المنطقة 25.3%. كانت نسبة 6% من الأشخاص ذات لُطاخة بلغم إيجابية تُؤكد الإصابة بالسل من بين مجموع الحالات التي تم خضوعها للفحص بواسطة المجهر. بلغت نسبة نجاح العلاج 91.8% محليا وهي نسبة مرتفعة مقارنة بالمستوى الإقليمي بنسبة 85% (كوهورت 2014).

الخاتمة: أكدت هذه الدراسة على سهولة القيام بعملية دراسة الحالات من قبل المجموعات المتنقلة في المناطق التي يصعب الوصول إليها وخاصة عندما يتم تجهيزهم بالأجهزة اللازمة المحمولة كجهاز التصوير الشعاعي الرقمي للصدر والتي تُقدّم نتائج فورية. ولكن شكّلت عملية المتابعة للفحص المجهري للبلغم حاجزا ملحوظا لتأكيد نتائج التشخيص. وبهدف تحسين عملية دراسة الحالات استنادا إلى أنشطة المجموعات المتنقلة ، ينبغي تعزيز تعدد الخدمات في المستقبل بما في ذلك التشخيص الجزيئي أو توفير كؤوس أخذ عينات البلغم لجميع الحالات الافتراضية للإصابة بالسل قبل القيام بالتصوير الشعاعي الرقمي للصدر واختبار ما إذا كان التصوير الرقمي الشعاعي للصدر يدل على الإصابة بالسل.

Translated from English version into Arabic by Zeineb TRABELSI, through

TRANSLATORS WITHOUT BORDERS

在缅甸通过移动团队主动发现肺结核病例的产出和治疗结果

Ohnmar Myint, Saw Saw, Petros Isaakidis, Mohammed Khogali, Anthony Reid, Nguyen Binh Hoa, Thi Thi Kyaw, Ko Ko Zaw, Tin Mi Mi Khaing, Si Thu Aung

摘要

引言：自2005年起，缅甸国家肺结核项目(NTP)开始依靠移动团队在难以到达地区实施主动发现病例(ACF)行动。本研究揭示了移动团队在总肺结核病例检测中的贡献，移动团队检测的肺结核患者特征及其治疗结果。
方法：本研究是一项针对2014年10月-12月常规项目数据的描述性研究。移动团队的活动为一站式服务，包括移动数字胸片(CXR)和两份痰标本的显微镜镜检。病例检查的流程为根据临床症状筛查，再通过移动数字胸片(CXR)和痰标本显微镜镜检确认。确诊病例开始治疗直至确定最终结果。
结果：共有9 349名有肺结核症状人员（占纳入研究人员的96.6%）进行了数字胸片筛查。其中，这些人员中的51.6%进行了痰液检查。最终，移动团队确诊了504例肺结核患者，占到各乡镇总病例的25.3%。显微镜镜检的病例中，6%的为涂片阳性。本研究的乡镇治疗成功率（91.8%）比2014年全国治疗成功率（85%）高。
结论：本研究证实了在难以到达地区移动团队主动发现病例的可行性和可接受性，特别是当配备了能够提供快速诊断结果的手提式数字胸片机。然而，痰液检查的后续检查成为确诊的重大障碍。为了进一步优化移动团队主动发现病例，将来的一站式移动团队主动发现病例活动应继续加强，包括提供分子诊断和在数字胸片检查前对所有的假定肺结核病例提供痰罐并在数字胸片有提示的情况下进一步测试。

Translated from English version into Chinese by Xin-Yu Feng, edited by Pin Yang

CDC CHINESE CENTER FOR DISEASE CONTROL AND PREVENTION
NATIONAL INSTITUTE OF PARASITIC DISEASES

Identification active de cas de tuberculose par des équipes mobiles au Myanmar : rendement et résultats thérapeutiques

Ohnmar Myint, Saw Saw, Petros Isaakidis, Mohammed Khogali, Anthony Reid, Nguyen Binh Hoa, Thi Thi Kyaw, Ko Ko Zaw, Tin Mi Mi Khaing, Si Thu Aung

Résumé

Contexte: depuis 2005, le Programme national de lutte contre la tuberculose du Myanmar met en œuvre des activités d'identification active de cas réalisées par des équipes mobiles dans des régions difficiles d'accès. La présente étude a révélé la contribution des activités des équipes mobiles à la détection globale des cas de tuberculose (TB), à la détermination des caractéristiques de patients atteints de la TB détectées par les équipes mobiles et à leurs résultats thérapeutiques.
Méthodes: il s'agit d'une étude descriptive reposant sur des données de programme de routine collectées entre octobre et décembre 2014. Les activités des équipes mobiles constituaient un service unique et impliquaient la réalisation d'une radiographie pulmonaire numérique portable et l'examen microscopique de deux échantillons d'expectorations. L'algorithme de détection des cas comprenait le dépistage de patients en fonction des symptômes, puis la réalisation d'une radiographie pulmonaire numérique portable suivie d'un examen d'expectorations par microscopie à des fins de confirmation. Le traitement des patients diagnostiqués a été lancé et poursuivi jusqu'à ce que le résultat final ait été déterminé avec certitude.

Résultats: au total, 9349 personnes présentant des symptômes suggérant une TB ont subi un dépistage par radiographie pulmonaire, ce qui représente un taux d'adhésion de 96,6 %. Sur le nombre total de personnes devant subir un examen des expectorations par microscopie, 51,6 % d'entre elles ont fait examiné leurs expectorations. Pour finir, 504 patients atteints de TB ont été identifiés par les équipes mobiles et la contribution totale à la détection de cas de TB dans les municipalités respectives s'élevait à 25,3 %. Parmi l'ensemble des cas examinés par microscopie, 6 % des cas présentaient un résultat d'examen des expectorations positif pour la TB. Le taux de réussite thérapeutique atteignait 91,8 % au sein des municipalités étudiées par rapport au taux national de 85 % (cohorte de 2014).

Conclusions: la présente étude a confirmé la faisabilité et l'acceptabilité d'activités d'identification active de cas par des équipes mobiles dans des régions difficiles d'accès, notamment lorsque ces dernières sont équipées de machines de radiographie portables fournissant des résultats immédiats. Le processus de suivi de l'examen des expectorations constituait néanmoins un obstacle significatif à la confirmation du diagnostic. Afin d'optimiser les activités d'identification active de cas par des équipes mobiles, les futures activités d'identification active de cas étaient nécessaires pour renforcer le service unique comprenant le diagnostic moléculaire ou la fourniture de gobelets de recueil d'expectorations à tous les cas de TB supposés préalablement à la radiographie pulmonaire et à l'analyse de la radiographie pulmonaire afin de déterminer si le cliché suggère la présence de la TB.

Translated from English version into French by eric ragu, through

TRANSLATORS
WITHOUT BORDERS

Активное выявление случаев заболевания туберкулёзом передвижными группами в Мьянме: показатели обнаружения и лечения

Охнмар Майинт (Ohnmar Myint), Сау Сау (Saw Saw), Петрос Исаакидис (Petros Isaakidis), Мохаммед Хогали (Mohammed Khogali), Энтони Рейд (Anthony Reid), Нгуен Бинь Хоа (Nguyen Binh Hoa), Фи Фи Чиао (Thi Thi Kyaw), Ко Ко Зау (Ko Ko Zaw), Тин Ми Ми Кхайн (Tin Mi Mi Khaing), Си Ту Аун (Si Thu Aung)

Аннотация

Краткое описание. Начиная с 2005 года, в Национальную программу Мьянмы по борьбе с туберкулёзом входят меры по активному выявлению заболеваний (АВЗ), которые включают отправку передвижных групп в труднодоступные районы. В данном исследовании сообщаются результаты вклада передвижных групп в общее число выявленных заболеваний туберкулёзом (ТБ); характеристики больных ТБ, обнаруженных передвижными группами, и результаты их лечения.

Методы. В этом описательном исследовании использованы стандартные данные программы, полученные с октября по декабрь 2014 г. Передвижные группы оказывали комплексное обслуживание в едином центре, в которое входили портативная цифровая рентгенография

грудной клетки (РГК) и микроскопия двух образцов мокроты. Процедура выявления заболеваний заключалась в отборе пациентов на основании симптомов, затем обследовании с помощью РГК, после которого выполнялась микроскопия мокроты для подтверждения диагноза. Больные с установленным ТБ начинали лечение и продолжали получать его до тех пор, как был обеспечен окончательный результат.
Результаты. Общее количество пациентов с симптомами ТБ, прошедших РГК с величиной накопления 96,6%, составило 9349 человек. Из числа тех, кому была необходима микроскопия мокроты, 51,6% пациентов сделали этот анализ. В конечном счёте, передвижными группами были обнаружены 504 больных ТБ, и их итоговый вклад в общее число выявленных заболеваний ТБ в соответствующих населённых пунктах составил 25,3%. Среди всех случаев, прошедших проверку микроскопией, 6% мазков были положительными на ТБ. Показатель успешности лечения в населённых пунктах исследования достиг 91,8% по сравнению с показателем по стране 85% (данные за 2014 год).
Заключение. Данное исследование подтвердило выполнимость и приемлемость проведения АВЗ передвижными группами в труднодоступных районах, особенно если они снабжены портативными цифровыми устройствами РГК, которые дают немедленные результаты. Однако последующая процедура анализа мокроты явилась значительным препятствием для подтверждения диагноза. Для оптимизации АВЗ с использованием передвижных групп необходимо сделать будущие мероприятия АВЗ еще более комплексными за счёт обеспечения в едином центре обслуживания всех пациентов с подозрением на ТБ молекулярной диагностикой или ёмкостями для сбора мокроты до выполнения РГК и тестирования, если РГК указывает на ТБ.

Translated from English version into Russian by Natalia Potashnik, through

TRANSLATORS
WITHOUT BORDERS

Búsqueda activa de casos de tuberculosis por parte de equipos móviles en Myanmar: rentabilidad del diagnóstico y resultados del tratamiento

Ohnmar Myint, Saw Saw, Petros Isaakidis, Mohammed Khogali, Anthony Reid, Nguyen Binh Hoa, Thi Thi Kyaw, Ko Ko Zaw, Tin Mi Mi Khaing, Si Thu Aung

Resumen

Información previa: Desde 2005, el Programa Nacional de Lucha contra la Tuberculosis de Myanmar (NTP, en inglés) viene implementando actividades de búsqueda activa de casos (ACF, en inglés) en las cuales participan equipos móviles para llegar a zonas de difícil acceso. Este estudio reveló la contribución de las actividades de los equipos móviles a la detección de la totalidad de los casos de tuberculosis (TB), las características de los pacientes con TB detectados por los equipos móviles y los resultados de su tratamiento.

Métodos: Este fue un estudio descriptivo en el que se utilizaron datos de programas de rutina entre octubre de 2014 y diciembre de 2014. Las actividades del equipo móvil eran un servicio todo en uno, que incluían radiografía de tórax digital portátil y microscopía de dos muestras de esputo. El algoritmo de la detección de casos incluía la selección de pacientes por síntomas, luego radiografía de tórax y finalmente una microscopía de esputo para confirmar el diagnóstico. Los pacientes diagnosticados comenzaron un tratamiento y se les hizo un seguimiento hasta verificar el resultado final.
Resultados: Un total de 9 349 personas con síntomas que parecían indicar la presencia de TB fueron evaluadas con una radiografía de tórax. El 96,6 % fueron admitidos. De aquellos que debían someterse a una microscopía de esputo, se examinó el esputo del 51,6 %. Finalmente, los equipos móviles identificaron 504 pacientes con TB y la contribución general a la detección total de casos de TB en las localidades respectivas fue del 25,3 %. De todos los casos examinados por microscopía, el 6 % dieron positivo para TB en el frotis de esputo. La tasa de éxito del tratamiento llegó a ser del 91,8 % en las localidades del estudio en comparación con la tasa nacional del 85 % (cohorte 2014).
Conclusiones: Este estudio confirmó la viabilidad y aceptabilidad de la búsqueda activa de casos por parte de equipos móviles en contextos de difícil acceso, especialmente cuando estos estaban equipados con máquinas para radiografía de tórax portátiles que permitieron obtener resultados inmediatos. Sin embargo, el proceso de seguimiento del examen de esputo fue un obstáculo considerable para confirmar el diagnóstico. A fin de optimizar la búsqueda activa de casos mediante la actividad de equipos móviles, las actividades futuras de búsqueda activa de casos deben reforzarse con un servicio todo en uno que incluya el diagnóstico molecular o la provisión de recipientes para esputo a todos los casos presuntos de TB antes de la radiografía de tórax y así poder realizar análisis si la radiografía sugiere la presencia de TB.

Translated from English version into Spanish by María Emilia Meini, through

TRANSLATORS
WITHOUT BORDERS
